# Computational Modeling of Macrophage Iron Sequestration during Host Defense against Aspergillus

**DOI:** 10.1128/msphere.00074-22

**Published:** 2022-07-12

**Authors:** Bandita Adhikari, Yogesh Scindia, Luis Sordo Vieira, Henrique de Assis Lopes Ribeiro, Joseph Masison, Ning Yang, Luis L. Fonseca, Matthew Wheeler, Adam C. Knapp, Yu Mei, Brian Helba, Carl Atkinson, Will Schroeder, Borna Mehrad, Reinhard Laubenbacher

**Affiliations:** a University of Pennsylvania, Philadelphia, Pennsylvania, USA; b Center for Quantitative Medicine, University of Connecticut Health Center, Farmington, Connecticut, USA; c University of Florida, Gainesville, Florida, USA; d Kitware Inc., Albany, New York, USA; University of Georgia

**Keywords:** iron regulation, *Aspergillus fumigatus*, macrophage, mathematical model

## Abstract

Iron is essential to the virulence of Aspergillus species, and restricting iron availability is a critical mechanism of antimicrobial host defense. Macrophages recruited to the site of infection are at the crux of this process, employing multiple intersecting mechanisms to orchestrate iron sequestration from pathogens. To gain an integrated understanding of how this is achieved in aspergillosis, we generated a transcriptomic time series of the response of human monocyte-derived macrophages to Aspergillus and used this and the available literature to construct a mechanistic computational model of iron handling of macrophages during this infection. We found an overwhelming macrophage response beginning 2 to 4 h after exposure to the fungus, which included upregulated transcription of iron import proteins transferrin receptor-1, divalent metal transporter-1, and ZIP family transporters, and downregulated transcription of the iron exporter ferroportin. The computational model, based on a discrete dynamical systems framework, consisted of 21 3-state nodes, and was validated with additional experimental data that were not used in model generation. The model accurately captures the steady state and the trajectories of most of the quantitatively measured nodes. In the experimental data, we surprisingly found that transferrin receptor-1 upregulation preceded the induction of inflammatory cytokines, a feature that deviated from model predictions. Model simulations suggested that direct induction of transferrin receptor-1 (TfR1) after fungal recognition, independent of the iron regulatory protein-labile iron pool (IRP-LIP) system, explains this finding. We anticipate that this model will contribute to a quantitative understanding of iron regulation as a fundamental host defense mechanism during aspergillosis.

**IMPORTANCE** Invasive pulmonary aspergillosis is a major cause of death among immunosuppressed individuals despite the best available therapy. Depriving the pathogen of iron is an essential component of host defense in this infection, but the mechanisms by which the host achieves this are complex. To understand how recruited macrophages mediate iron deprivation during the infection, we developed and validated a mechanistic computational model that integrates the available information in the field. The insights provided by this approach can help in designing iron modulation therapies as anti-fungal treatments.

## INTRODUCTION

The incidence of invasive aspergillosis continues to grow in tandem with the increasing use of immunosuppressive therapies (1, 2). Despite advances in diagnosis and therapy, the mortality rate of invasive aspergillosis remains 30–60%, with most deaths occurring in patients on the best available therapy (3–5). The increasing prevalence of triazole resistance in this infection (6, 7) has raised the specter of a “perfect storm” due to a growing population of susceptible individuals with a diminished repertoire of treatment options (8).

Nutritional immunity, broadly defined as the restriction of essential nutrients from invading pathogens (9, 10), is an important component of antimicrobial host defenses (11, 12). The battle over iron represents the best-defined example of nutritional immunity (13–17) and is highly relevant to aspergillosis; iron overload is an independent risk factor for invasive aspergillosis (18), and iron acquisition is essential to virulence of Aspergillus species (19–21). The host sequestration of iron during the infection is implemented via multiple interrelated dynamic mechanisms, including cellular uptake of iron and heme, intracellular iron storage, and systemic suppression of iron availability, but the interplay of these host mechanisms during the infection is highly complex and poorly defined.

Tissue macrophages are central to local control of iron availability, both under homeostatic conditions and after tissue injury (22). At baseline, the normal lung of both mice and humans contain two populations of macrophages with different lineages: alveolar macrophages, which are yolk sac-derived, self-renewing, and long-lived cells residing on the luminal side of the epithelium (23); and monocyte-derived macrophages, which are short-lived and constantly replenished by circulating monocytes, residing on the abluminal side of the epithelium and referred to as interstitial macrophages under baseline conditions (24, 25). The monocyte-derived macrophage population greatly expands within hours of Aspergillus challenge due to CCR2-mediated recruitment of classical monocytes, which cross the epithelium, interact with swollen conidia and early germlings, and are essential to host defense against Aspergillus (26).

Mathematical modeling is a powerful tool for a principled integration of biological data and mechanisms, and the generation of novel hypotheses. While the response of activated macrophages to other lung infections has been studied using mathematical models (27–31), the published models do not address iron-related nutritional immunity in the setting of infection. Similarly, mathematical models of systemic iron regulation, macrophage iron-handling, and iron metabolism during erythropoiesis (32–34) are not specific to infections. Our group previously built a computational model of the competition of host immune cells and Aspergillus for access to iron during invasive infection (35), but this model did not include the intracellular handling of iron in macrophages in response to the infection. The focus of the current study is therefore to construct such a model and use it as a tool to integrate macrophage iron homeostasis upon contact with the fungus.

Here, we report the development of a novel mechanistic model of macrophage iron-handling during macrophage interaction with Aspergillus conidia that quantitatively captures the molecular events in macrophage iron regulatory pathways over time. We generated a longitudinal transcriptomics data set from human monocyte-derived macrophages infected with Aspergillus fumigatus conidia and used it, together with available information from the literature, to construct a mathematical model that integrates pathogen recognition, transcriptional and posttranscriptional regulation, and autocrine/paracrine feedback loops influencing macrophage iron import, export, and storage. We validated this model with independent experimental data. The model was found to reproduce the dynamic changes in macrophage iron handling that were observed experimentally, which work in concert to limit the extracellular iron pool.

## RESULTS

### Transcriptomic analysis of macrophage-Aspergillus interaction shows activation of major innate immune pathways, including iron regulation.

**FIG 1:**
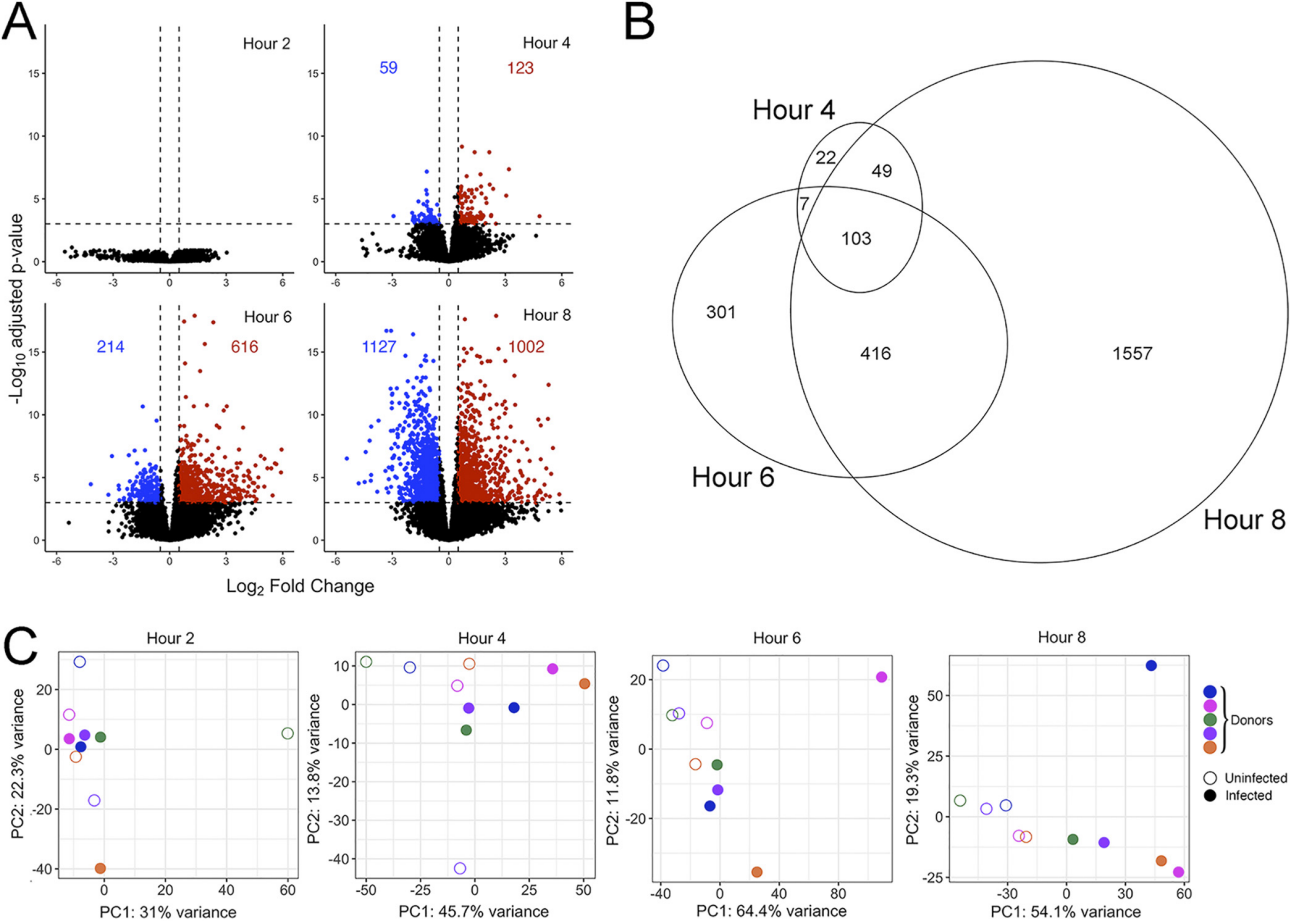
Differential expression analysis of macrophages infected with Aspergillus. (A and B) Volcano plots and Euler diagram of genes with |Log_2_ fold change| ≥ 0.5 differential expression in infected compared to uninfected macrophages with adjusted *P* value < 0.001. The number of differentially regulated genes is indicated in each panel. (C) Principal-component analysis plots of read counts of differentially expressed genes at each time point, after variance stabilizing transformation. Open and filled symbols indicate uninfected and infected cells, respectively, and the color of symbols denotes the donor.

We began with a time series experiment to measure the transcriptional landscape of human monocyte-derived macrophages that were incubated alone or with A. fumigatus conidia over 8 h. We observed a time-dependent increase in the number of differentially expressed genes between macrophages incubated alone compared to those cocultured with A. fumigatus, beginning 4 h after incubation (Fig. 1A and B), consistent with the timing of shedding of the conidial rodlet layer and surface expression of the dectin-1 ligand (13), b-d-glucan (36). Principal-component analysis of normalized read counts of the top 500 differentially expressed genes showed separation of infected and uninfected samples at 6 and 8-h time points (Fig. 1C).

**FIG 2:**
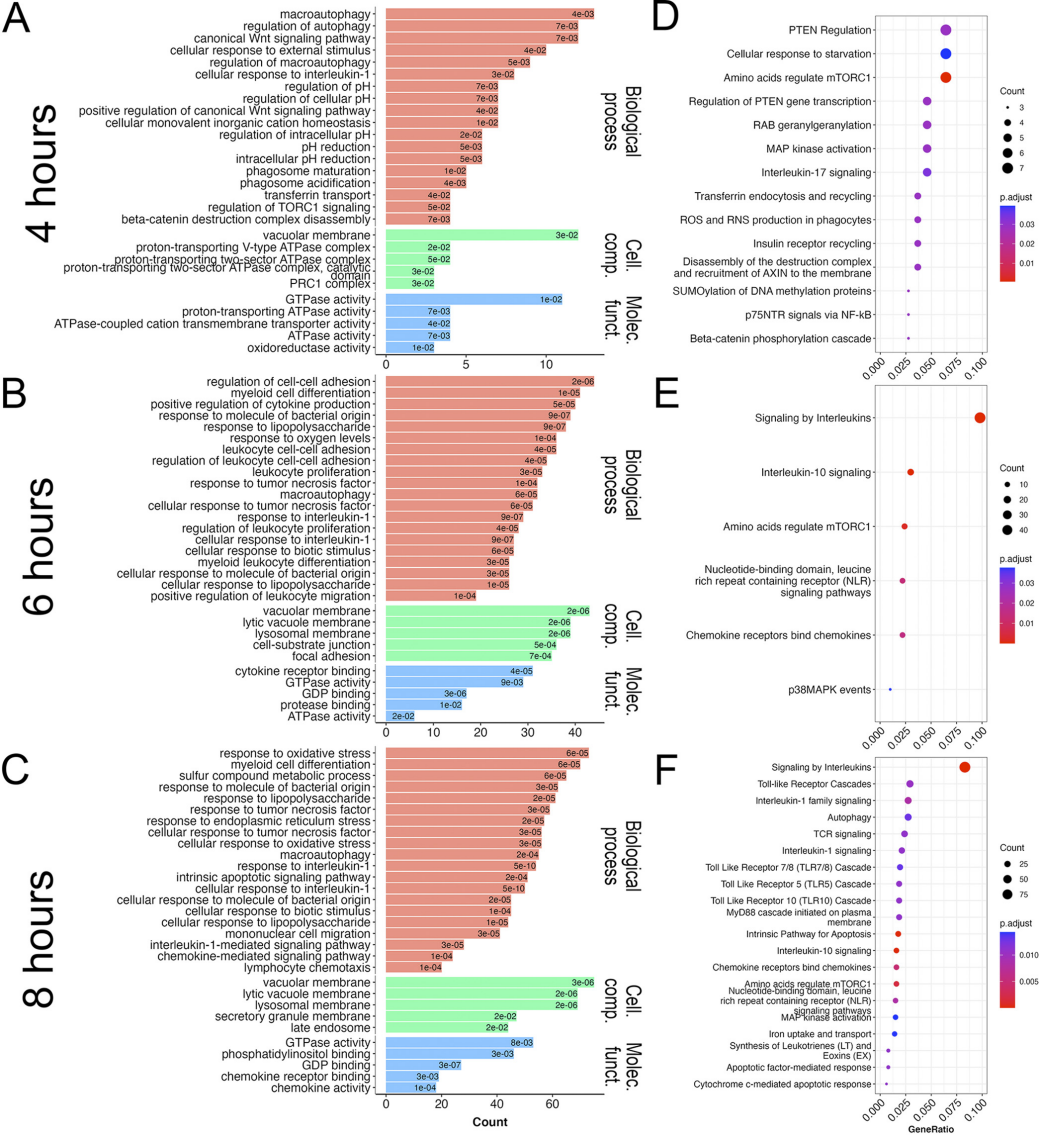
Enrichment analysis of differentially expressed genes in macrophages infected with Aspergillus. GO terms (A–C) and Reactome pathways (D–F) at 4, 6, and 8 h after infection, respectively. Enrichment analysis was performed with differentially expressed genes (adjusted *P* value <0.001 and |Log_2_ fold change| ≥ 0.5). Enriched terms for Gene ontology (top 20 for biological processes, and top 5 for cellular components and molecular functions) and Reactome pathways (top 20) are reported. Cell. comp., cellular component; GeneRatio, the ratio of the number of enriched genes in a given pathway to the total number of genes in that pathway; Molec. funct., molecular function.

We next performed enrichment analysis of differentially expressed genes at 4, 6, and 8 h to obtain enriched Gene Ontology terms for biological processes, molecular functions and cellular components, and to identify related Reactome pathways. At 4 h, we observed gene sets enriched for biological processes relating to phagocytosis (such as “phagosome acidification,” “endocytosis,” and “regulation of intracellular pH”) and, notably, “transferrin transport” (Fig. 2A). Consistent with these observations, “transferrin endocytosis and recycling” and “ROS and RNS production in phagocytosis” pathways were enriched for Reactome analysis at 4 h (Fig. 2B). Differential genes at 6 and 8 h were enriched for Gene Ontology terms relating to multiple immune processes (e.g., “cellular response to lipopolysaccharides,” “response to tumor necrosis factor,” “cellular response to interleukin-1,” “neutrophil chemotaxis,” and “cell-cell signaling”; Fig. 2B and C) and iron transport processes (e.g., “transferrin transport” and “iron transport”). Reactome pathways analysis further confirmed activation of immune pathways with the enrichment of “NF-κB signaling pathway,” “TNF signaling pathway,” “cytokine-cytokine receptor pathway,” and “chemokine signaling pathway” at 6 and 8 h (Fig. 2E and F). We also observed that the “Iron uptake and transport” pathway was enriched at 8 h, with 16 differentially regulated genes present in the pathway (Fig. 2F).

**FIG 3:**
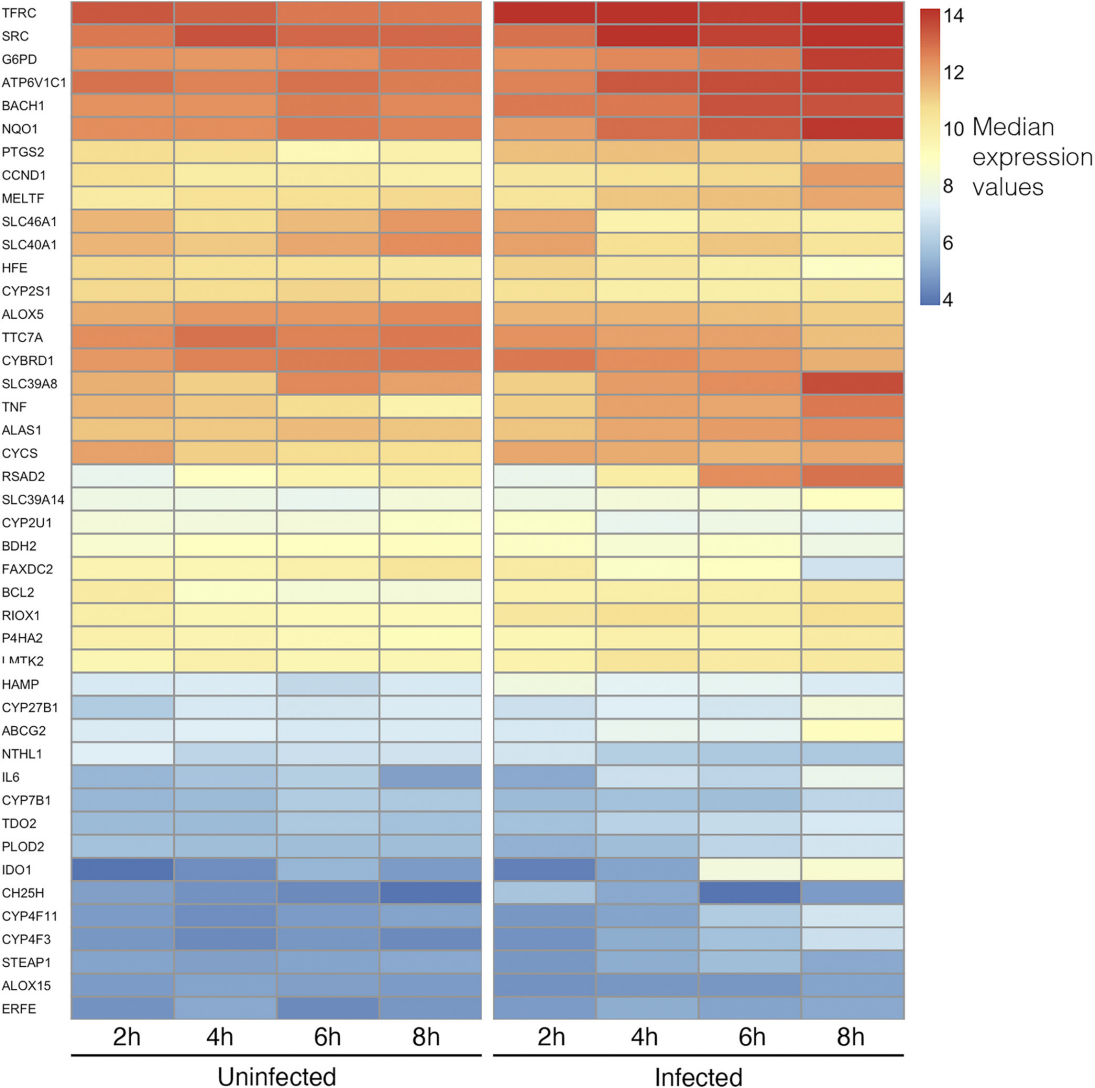
Heatmap of differentially regulated iron-associated genes after unsupervised clustering. Heatmap showing treatment groups on the *x* axis and differentially regulated iron-associated genes with a |Log_2_ fold change| ≥ 1 on the *y* axis. Each cell represents the median expression value of 5 biological replicates after variance stabilizing transformation on size factor normalized count data.

We next focused on enrichment of iron-related genes. Using Gene Ontology and Reactome analyses, we observed pathways operational in iron regulation, including transferrin transport, iron transport, tumor necrosis factor (TNF)- and interleukin (IL)-1-signaling. Ingenuity Pathway Analysis (IPA) also indicated that, out of all “iron homeostasis” network molecules present in the IPA database, 26 genes involved in iron import/storage/export/transport pathways were differentially expressed. Unsupervised clustering of average expression data of iron-related genes obtained from AmiGO-2 revealed markedly different expression patterns for the 4, 6, and 8 h infected culture groups, compared to control and 2-h infected samples (Fig. 3). Overall, these data indicated early and robust activation of iron regulatory mechanisms in macrophages after fungal detection.

### Mathematical model of iron regulation in macrophages.

**FIG 4:**
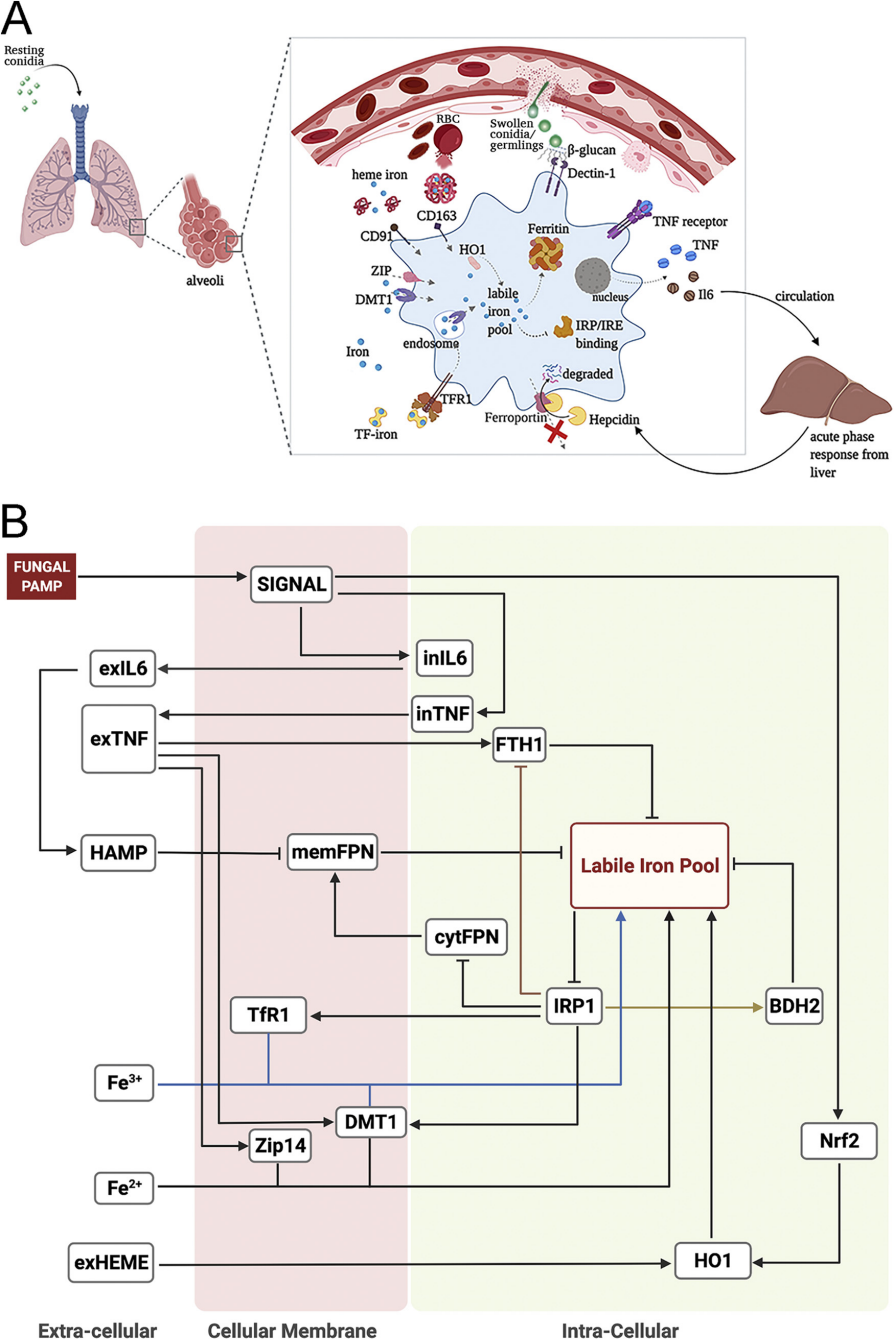
Computational model created with BioRender.com. (A) Diagrammatic representation of key processes in iron regulation in macrophages during invasive pulmonary aspergillosis. (B) Wiring diagram of macrophage iron regulation during invasive pulmonary aspergillosis (see Table 2 for references). Pointed arrows represent activation and blunt arrows represent inhibition. Some arrows are colored for better visualization. Extracellular, membrane, cytoplasm, and intracellular molecules are indicated by ex-, mem-, cyt-, and in- prefixes. BDH2, 3-hydroxybutyrate dehydrogenase-2; DMT1, divalent metal transporter-1; Fe^2+^, ferrous iron forms; Fe^3+^, ferric iron forms; FPN, ferroportin; FTH1, ferritin heavy-chain-1; HAMP, hepcidin; HO1, heme oxygenase-1; IL-6, interleukin-6; LIP, labile iron pool; IRP1, iron-regulatory protein-1; PAMP, pathogen-associated molecular pattern; TfR1, transferrin receptor-1; TNF, tumor necrosis factor; Zip14, zinc transporter-14.

We next integrated the RNA-seq results above with known biology to construct a mathematical model of iron regulation in monocyte-derived macrophages during an encounter with Aspergillus conidia. We reviewed the literature on each of the iron-related genes identified in our data and assessed their relevance to iron regulation and handling during fungal infections. We then established a set of molecules to include in the model (Table 1), based on our data and known literature (described below and depicted in Fig. 4A). These molecules were incorporated into a static network (Fig. 4B), which formed the basis for a discrete dynamical model, with each node in the model taking on three possible discrete states, 0, 1, and 2. Hence, a model state is described as a vector of length 21 (the number of variables in the model), with entries 0, 1, or 2, representing different levels of each molecule. From a given initial state, the model evolves in discrete time steps by applying the regulatory rules in Table 2.

**TABLE 1:** Biological description of variables and their possible states in the computational model

Node^*a*^	Name	Type	Location	Model states		
				0	1	2
BDH2	3-hydroxybutyrate dehydrogenase-2	Protein	Intracellular	Low expression	Normal	High expression
cytFPN	Cytoplasmic ferroportin	RNA	Intracellular	Low expression	Normal	High expression
DMT1	Divalent metal transporter-1	Protein, importer	Membrane	Low activity	Normal	High activity
exIL6	Interleukin-6	Cytokine	Extracellular	Low expression	Normal	High expression
exHeme	Heme	Compound, Molecule	Extracellular	Low concn	Normal	High concn
exTNF	Tumor necrosis factor	Cytokine	Extracellular	Low expression	Normal	High expression
Fe^2+^	Labile ferrous iron ions	Ion	Extracellular	Low concn	Normal	High concn
Fe^3+^	Transferrin-bound ferric iron ions	Ion	Extracellular	Low concn	Normal	High concn
FTH1	Ferritin heavy chain	Protein	Intracellular	Low expression	Normal	High expression
FUNGUS	A. fumigatus	Pathogen	Extracellular	Absent	Present	Present
HAMP	Hepcidin	Protein	Extracellular	Low concn	Normal	High concn
HO1	Heme oxygenase-1	Enzyme	Intracellular	Low expression	Normal	High expression
inIL6	Interleukin-6	Cytokine	Intracellular	Low expression	Normal	High expression
inTNF	Tumor necrosis factor	Cytokine	Intracellular	Low expression	Normal	High expression
IRP1	Iron regulatory protein	Protein	Intracellular	Low activity	Normal	High activity
LIP	Labile iron pool	Molecules	Intracellular	Low concn	Normal	High concn
memFPN	Membrane-bound ferroportin	Protein, Exporter	Membrane	Low expression	Normal	High expression
Nrf2	Nuclear factor erythroid factor 2-related factor 2	Transcription factor	Intracellular	Low concn	Normal	High concn
TfR1	Transferrin receptor-1	Protein, Importer	Membrane	Low activity	Normal	High activity
SIGNAL	PAMP signaling after recognition of pathogen	Pathway	Intracellular	Low activity	High activity	High activity
Zip14	Zrt- and Irt-like protein-14	Protein, Importer	Membrane	Low activity	Normal	High activity

a Extracellular, membrane, cytoplasm, and intracellular molecules are indicated by ex-, mem-, cyt-, and in- prefixes, respectively.

**TABLE 2:** Update rules of model species and supporting literature citations. Continuity function accounts for the previous state of the target molecule when changing the state of the target molecule from a high to low level.

Target*^a^*	Update rules^*a*^^*b*^	Description
BDH2	IRP	BDH2 has an IRE motif on its 3′ end. This interaction can lead to stabilization and increase in BDH2 (74).
cytFPN	cont(not(IRP1))	IRP1 can bind to the IRE present on the 5′ of ferroportin RNA inhibiting the translation of FPN (118).
DMT1	max(cont(exTNF), cont(IRP1))	TNF induces the expression of DMT1 during infection, and DMT1 has an IRE element on 3′end of its mRNA (43, 119).
exIL6	cont(inIL6)	IL6 is secreted into the extracellular environment (120).
exHeme	External Parameter	
exTNF	cont(inTNF)	TNF is secreted into the extracellular environment (120).
Fe^2+^	External Parameter	
Fe^3+^	External Parameter	
FTH1	max(cont(exTNF), cont(not(IRP)))	TNF induces the expression of FTH1, and FTH1 has an IRE element on the 5′ end of its mRNA for IRP regulation (43, 118, 121).
FUNGUS	Source Node	
HAMP	cont(exIL6)	Hepcidin is produced by the liver in response to the IL-6 (42, 93).
HO1	min(exHEME, cont(Nrf2))	Heme and NRF2 can activate expression of HO1 (122, 123).
inIL6	SIGNAL	IL6 is produced in response to fungus (37–39, 124, 125).
inTNF	SIGNAL	TNF is produced in response to fungus (38, 39, 41, 124, 125).
IRP1	cont(not(LIP))	IRE-binding activity of IRP1 is high in iron-deplete conditions (126).
LIP	cont(min(max(min(Fe^3+^, TfR1), min(Fe^2+^, DMT1, Zip14), HO1), min(not(memFPN), not(BDH2), not(FTH1)))	Import of transferrin-bound iron, free iron, and heme-iron increases intracellular iron. Storage of iron in ferritin, export of iron through ferroportin, and sequestration of iron by BDH2 decrease the labile iron pool in the cytosol (50).
memFPN	min(cont(cytFPN), not(HAMP))	Translated ferroportin locates to cell membranes. Membrane ferroportin can be targeted by hepcidin for degradation (49).
Nrf2	SIGNAL	NRF2 is produced in response to fungal beta-glucan (122).
TfR1	max(cont(IRP1), SIGNAL)	IRP1 stabilizes TfR1 mRNA by binding to the IRE element on the 3′ end of its mRNA thereby increasing total TfR1 (77, 127, 128).
SIGNAL	SIGNAL = low if FUNGUS =0, else SIGNAL = high	SIGNAL represents the activation of macrophages by the fungus(37).
Zip14	cont(exTNF)	TNF induces expression of Zip14 (43).

a Extracellular, membrane, cytoplasm, and intracellular molecules are indicated by ex-, mem-, cyt-, and in- prefixes, respectively.

b min, minimum; max, maximum; cont, continuity function.

Macrophages recognize fungal pathogen-associated molecular patterns via surface pathogen-recognition receptors (37–40), leading to the production and secretion of TNF and IL-6 (39, 41). We represent this recognition by the presence of FUNGUS and activation of SIGNAL (Fig. 4B). IL-6 induces the synthesis of hepcidin in the liver, a key iron regulatory hormone that is highly sensitive to systemic iron levels and, independently, inflammation (15, 42). TNF induces transcription of ferritin heavy chain-1 (FTH1), DMT1, and Zip14 via an autocrine/paracrine loop (43–47). Iron export occurs via the membrane protein ferroportin (48). Extracellular hepcidin binds to membrane ferroportin, mediating its internalization and subsequent proteolysis in endosomes, thereby lowering the efflux of iron to the extracellular environment (49, 50). We did not implement hepcidin production by macrophages in the model, because the effect has been reported to be comparatively negligible (51).

We incorporated three forms of extracellular iron import: Transferrin-bound iron (Fe^3+^), free labile iron (Fe^2+^), and heme-associated iron. The extracellular labile iron concentration will be exceedingly low (52) and is therefore not included. The concentrations of Fe^3+^, Fe^2+^, and heme-associated iron serve as external inputs (i.e., they are not regulated by other components of the model). Transferrin, the principal extracellular iron transport protein, binds ferric iron ions with high affinity and is internalized by receptor-mediated endocytosis via the transferrin receptor (53–56). Iron molecules then dissociate from transferrin and are shuttled into the cytosolic labile iron pool by endosomal membrane DMT1 (57). Similarly, labile iron can be taken up by the membrane proteins DMT1 or Zip14 from the extracellular environment and imported into the cytoplasm (46, 47, 58–61). With catabolism of hemoglobin (cell-free hemoglobin, resulting from infection-induced hemorrhage), there will be an increase in free iron and heme-bound iron (61–63). Free heme is complexed to hemopexin and taken up via CD91 (64, 65). We have represented this source of heme-associated iron as exHEME. Once imported, heme iron is converted to free iron by heme oxygenase-1 (HO1) and added to the labile iron pool, a redox-active form of iron present in the cytosolic environment (66, 67).

Excess iron in the cytosol is stored in ferritin, a 24-mer protein composed of light- and heavy-chain subunits (L- and H-ferritin, respectively) (68, 69). H-ferritin is also a ferroxidase enzyme, mediating the oxidation of ferrous to ferric iron for storage, and L-ferritin is important in the nucleation of ferric iron (70–72). We modeled FTH1 because it is transcriptionally regulated by inflammatory signals (45, 73). Cytosolic iron can also be bound by 2,5-dihydroxybenzoic acid (DHBA) molecules, also known as the mammalian siderophore (74, 75), represented in the model by BDH2, the enzyme that catalyzes the formation of 2,5-DHBA (75). The iron regulatory protein-1 regulates the intracellular labile iron pool by binding to iron responsive elements (IRE) of the untranslated 3′ and 5′ regions of mRNA of TfR1, DMT1, FPN, FTH1, and BDH2, thereby modulating the translation of iron storage, importer proteins, and exporter proteins (74, 76–79). Under low intracellular iron conditions, IRP1-IRE binding activity is high, inhibiting the translation of ferritins and FPN, and promoting the translation of TfR1, DMT1, and BDH2, with the opposite effect under iron-replete conditions (74, 77, 80).

### Computational model captures macrophage behavior during infection.

**FIG 5:**
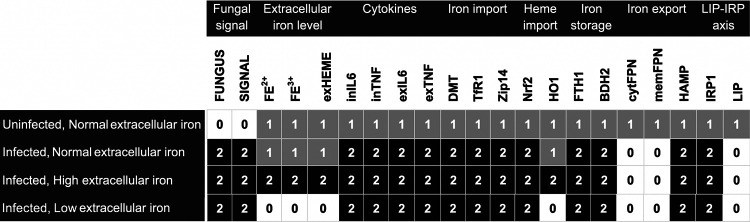
Different states of the computation model. Steady state simulations for the model under the conditions defined in Table 1 – uninfected macrophages in normal extracellular iron condition, and infected macrophages in normal, low, or high extracellular iron conditions. 0, low; 1, medium/normal; 2, high.

We next used the computational model to simulate macrophage behavior under uninfected conditions. The external input parameters of the model were fixed to normal values of 1, and model dynamics were explored through complete enumeration of all state transitions. The first row of Fig. 5 and Fig. S3 show that the model reached a steady state, corresponding to a physiologically normal state. To test the model behavior under infection, we next simulated the presence of A. fumigatus (FUNGUS=Present). This resulted in macrophage-activated intracellular signaling pathways and the production of IL-6, TNF, hepcidin, FTH1, Zip14, and DMT1. During infection, the model predicted low FPN transcription, membrane FPN, and the intracellular labile iron pool (LIP) in the steady state. Model simulation of an infected macrophage in the presence of high, normal, or low extracellular iron showed activation of HO1 to be dependent on extracellular heme concentrations, but did not affect the activation of other iron regulatory molecules (Fig. 5). HO1 catalyzes heme to ferrous iron, which adds to the intracellular labile iron pool (81–84) and is subsequently stored with ferritin.

10.1128/msphere.00074-22.4 FIG S3 Simulated steady state of the model output under conditions of exposure to the fungus and absent extracellular iron. Download FIG S3, TIF file, 0.7 MB. Copyright © 2022 Adhikari et al. 2022 Adhikari et al. https://creativecommons.org/licenses/by/4.0/ This content is distributed under the terms of the Creative Commons Attribution 4.0 International license.

### Validation of the computational model.

**FIG 6:**
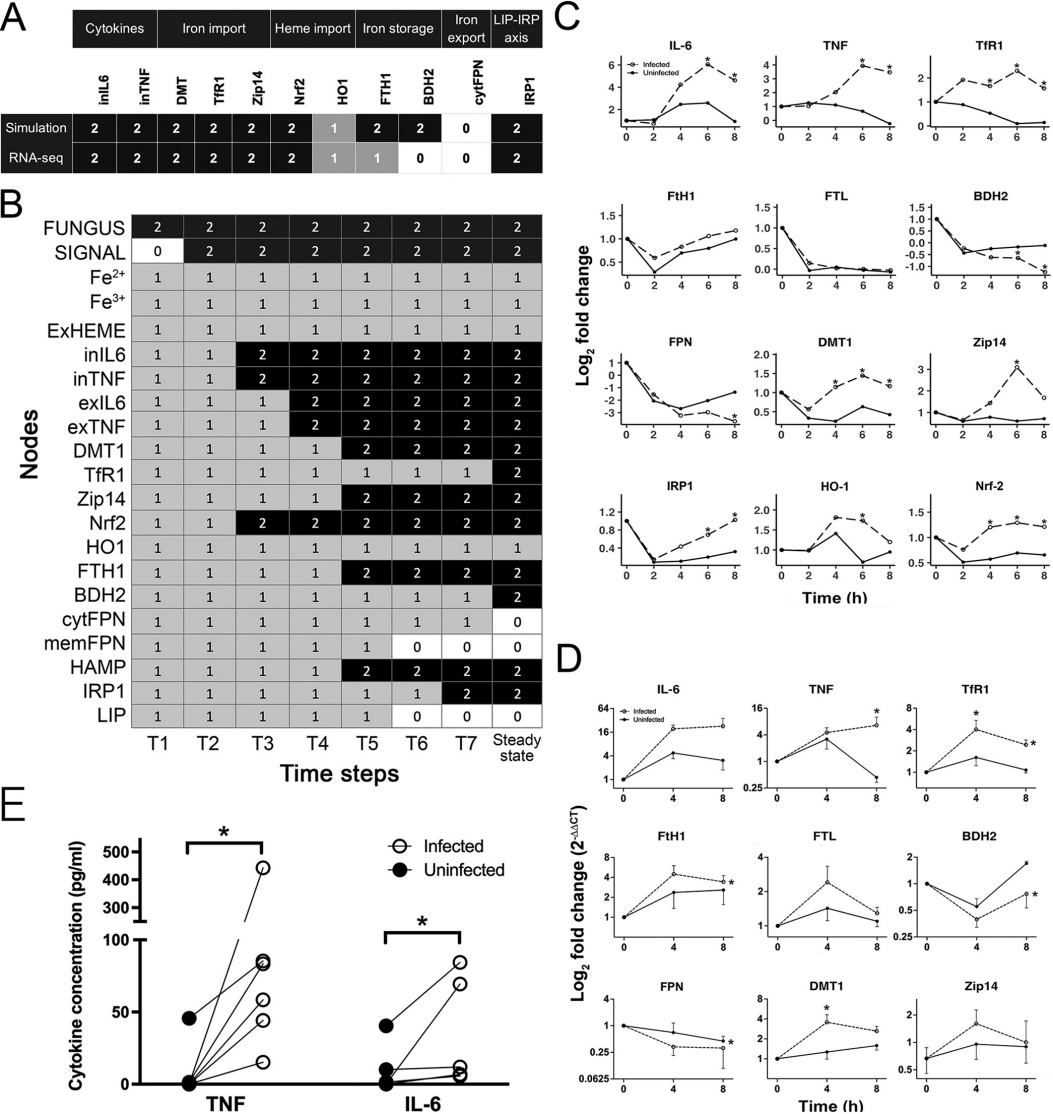
Validation of the computational model. (A) Simulated steady states for infected macrophages under normal extracellular iron level and the RNA-seq data at 8 h. Top row shows model output under conditions of exposure to the fungus and normal extracellular iron. Bottom row shows RNA-seq data discretized based on differential expression. (B) Simulated time-series of the model output under conditions of exposure to the fungus and absent extracellular iron. (C) RNA-seq experimental data was obtained from macrophage-Aspergillus cocultures without an external iron source. Read counts were normalized by the library size and a value of 0.5 was added to the normalized counts to generate pseudo counts, which were then transformed with a Log_2_ scale. Log-scaled reads are plotted against time and actual raw read counts, and the line was fitted with loess regression. Counts were plotted using DESeq2 function plotCounts method. *, *P* < 0.05, and the line was fitted to the data with loess regression. (D) Mean and SEM of qRT-PCR measurements from macrophages infected with Aspergillus. (E) Concentration of cytokines in supernatants of infected and uninfected macrophages after 8 h. Each line represents one donor. 0, downregulated; 1, no change; 2, upregulated. *, *P* < 0.05.

Model simulation showed that, for each of the three conditions we consider (uninfected, infected, and infected with high iron levels), the model reaches a distinct steady state. We experimentally validated the model and its dynamic behavior in two ways. First, we used the temporal evolution as exhibited by the RNA-seq data set described above, which was not used for model construction. There, we had only used a differential expression analysis over the entire time course. We compared the steady states obtained from model simulation with the experimental data at 8 h (Fig. 6A). Second, we compared the model trajectory to the temporal dynamics of our longitudinal transcriptomic data sets (Fig. 6B to D).

The model includes 11 intracellular nodes that are measurable at the transcription level, namely, TNF, IL-6, Zip14, TfR1, DMT1, FTH1, BDH2, IRP1, HO1, NRF2, and FPN. We found that the model steady state matched the experimental data for all nodes, except for BDH2 and FTH1 (Fig. 6A). BDH2 simulation shows increased expression whereas the experimental data shows decreased expression, as previously reported (85). This suggests that the model does not accurately capture BDH2 regulation, and that BDH2 is likely regulated by mechanisms independent of the known iron regulatory protein (IRP) regulation.

The model trajectory (Fig. 6B) matched the experimental data from the RNA-seq data set (Fig. 6C) for 10 of 12 nodes: The induction of IL-6 and NRF2 were observed in the simulation in earlier time steps and remained elevated until the steady state was reached, and both were differentially expressed from 4–8 h in the experiment. Similarly, the iron importers DMT1 and Zip14 were activated at early stages in model simulations, and differentially upregulated from 4–8 h in our experiment, suggesting induction of the iron import pathway post fungal recognition. Iron export, on the other hand, was inhibited upon the onset of infection in both simulation and in experimental data. The model indicated that membrane FPN is degraded at earlier time points whereas cytoplasmic FPN downregulation starts at later time steps in the simulation, suggesting that hepcidin regulation of FPN occurs prior to transcriptional regulation of FPN (Fig. 6B). Concurring with the simulation data, measured FPN transcripts (cytFPN) also showed downregulation only at 8 h in the experiment (Fig. 6C). We also simulated molecules relevant to iron storage and chelation, ferritin, and BDH2, respectively (Fig. 6B). We observed a slight change in FTH1 expression with no statistical significance and no change in ferritin light chain (FTL) expression (Fig. 6C). As a second step, experimental validation for these model predictions was performed on Aspergillus-infected macrophages using qRT-PCR and protein measurements (Fig. 6D and E). These measurements matched the model trajectory (Fig. 6B) for 8 out of 10 measured nodes and matched longitudinal transcriptome data (Fig. 6C) for all nodes.

### Mathematical modeling suggests macrophage iron regulatory responses precede, and are independent of, generation of inflammatory cytokines.

Mechanistic modeling can be used to assess whether known biology adequately accounts for observed behaviors. In our experimental data, we noted that the model accurately predicted the steady-state and temporal trajectory of TNF production, but while the model correctly predicted the steady-state TfR1 expression, it did not predict its trajectory correctly: In both the RNA-seq and qRT-PCR data sets, the upregulation of TfR1 occurred at 2–4 h, preceding that of TNF at 8 h, whereas the model predicted activation of TfR1 after the activation of IRP, indicating that the mechanisms incorporated into the model are not complete.

To assess this discordance, we tested different modifications of the model that would capture the trajectory of TfR1 expression matching the experimental observations. During fungal infections, nitric oxide has been reported to regulate TfR1, but our experimental data did not show expression of the iNOS gene, which is required for nitric oxide (NO) production in macrophages (86). We found that the only modification in the model that resulted in a steady state that reflected expected macrophage behavior during infection while maintaining all other trajectories and steady states was an additional regulation of TfR1 directly by the fungal node SIGNAL. With this modification, the model captures the observed phenotypic behavior in the experimental data. While TNF is one of the main cytokines operational in iron regulation during infection, early activation of TfR1, independent of IRP, suggests a separate activation pathway for enhancing iron import. The phenomenological regulation from SIGNAL to TfR1 suggests that further study of TfR1 regulation is needed to understand the mechanism of TfR1 induction during infection.

## DISCUSSION

Multicellular hosts have evolved an evolutionarily ancient system of iron regulation in order to deprive invading pathogens of this essential nutrient. In response to diverse inflammatory and infectious stimuli, this system mediates iron sequestration within macrophages; a precipitous fall in plasma iron, plasma transferrin, and transferrin saturation; and increase in plasma ferritin (10). Monocyte-derived macrophages recruited to the site of infection are at the crux of this system, controlling both the intracellular and extracellular iron availability via modulation of iron import, storage, and export mechanisms, in response to a combination of signals from iron availability, pathogen recognition, inflammatory cytokines, and systemic hormones. These processes are intricately interdependent and are thus difficult to study in isolation, but can be integrated and understood using mechanistic computational modeling. We integrated the existing literature on macrophage iron control during fungal infection and transcriptional data obtained from coculturing human monocyte-derived macrophages (representing lung interstitial macrophages and recruited inflammatory macrophages) with fungal cells to develop such a model as related to aspergillosis.

The differential gene expression analysis of macrophages cocultured with A. fumigatus revealed a transcriptional response that began between 2 and 4 h after exposure to the fungus, consistent with prior work (87–90). Activated genes included those related to iron transport, storage, binding, and reductases, indicating the activation of iron regulatory mechanisms. In our experimental system, Aspergillus had access only to the small concentration of iron contained in the culture media and intracellular iron of killed macrophages. Analogously, inhaled Aspergillus conidia only have access to the iron-poor alveolar fluid and iron released from necrotic cells (91). But as the infection progresses, Aspergillus gains access to iron from tissue hemorrhage and hemoglobin catabolism in the host – a circumstance not represented in the experimental coculture system, which models only the first 8 h of the infection. Our model simulations were validated with experimental data and reflect the expected biology of Aspergillus-infected macrophages under different extracellular iron levels.

Macrophages can sequester iron via three mechanisms: increased iron import, increased iron storage, and decreased iron export. Regulation of iron export from macrophages (as well as duodenal enterocytes), mediated by the hepcidin-ferroportin axis, has been extensively documented in the literature in the context of normal iron homeostasis and during infections (92, 93). Our model shows that upregulation of iron importers in response to fungal detection plays an essential role in macrophage iron sequestration during infection, independent of iron export. The induction of transferrin receptor-1 and DMT1 in the experimental data validated this finding. In this context, iron regulation in macrophages was previously studied with a coculture experiment of a RAW264.7 immortalized murine macrophage cell line (94). These cells showed an induction of ferritin, and a reduction of ferroportin expression after 7 h of coculture, but no change in TfR1 expression. The discrepancy between our findings and these results is likely due to differences between the murine cell line and primary human monocyte-derived macrophages, particularly the fact that RAW264.7 cells, derived from BALB/c mice, carry a homozygous non-functional NRAMP1 mutation, blocking iron shuttling between the phagosome and cytosol and impairing their iron homeostasis (95, 96).

Infection has been shown to result in reduction of extracellular iron to extremely low concentrations and in sequestration of iron inside macrophages (97–99). Our model simulation suggests that during infection, regardless of extracellular iron levels, the intracellular iron is stored by ferritin (Fig. 5). Our experimental data (Fig. 6C and D), however, showed a small upregulation of FTH1 and no change in FTL. Of note, the baseline expression of FTH1 in both the infected and uninfected conditions was high, and we speculate that a slight upregulation in expression is enough to store a large quantity of iron, since one ferritin molecule can store up to 4,500 iron ions (100). The model also elucidates the interplay between immune activation and the IRP-LIP axis of iron regulation: In Fig. 5, the model indicated that normal iron-homeostasis under physiologic conditions is perturbed during infection, with high expression of DMT1, Zip14, TfR1, and ferritin mediated by inflammatory signals (IL-6 and TNF) and independently of the IRP-LIP axis, resulting in augmented uptake and storage of iron in macrophages during infection. Consistent with this model prediction, the overriding influence of proinflammatory cytokines on iron regulation has been reported in other disease models. During inflammation in neuronal cells, the IL-6/JAK2/STAT3 pathway overrides iron homeostasis by dysregulation of hepcidin expression (101). In a study of the human monocytic cell line U937, TNF, IFN-ɑ, and IL-1β modulate iron metabolism by affecting macrophage iron uptake, TfR1 expression, intracellular iron handling, and ferritin mRNA levels (102).

Our computational model allowed us to capture the steady states of most model constituent molecules. Further analysis, however, indicated that the model did not capture some of the experimental observations. Particularly, it did not capture the surprising biological observation of the activation of TfR1 at an earlier time point than TNF, both in the RNA-seq experiment and qRT-PCR. We modified the model to capture this feature through the inclusion of a hypothetical mechanism that activated TfR1 from a fungal signal. With this modification, the model agreed with the experimental data, suggesting a new hypothesis of TfR1 activation by an unknown molecule upstream of TNF. The transcriptional and posttranscriptional regulation of TfR1 by cellular iron deficiency and hypoxia, via the HIF-HRE and the IRP-IRE systems, is well-described (103), but its direct regulation by infectious stimuli has not, to our knowledge, been documented. Interestingly, TfR1 has been shown to localize to the early endosome of macrophages within minutes after phagocytosis of Aspergillus conidia (104), a timeline consistent with our experimental results and revised model predictions on the transcriptional activation of TfR1.

We recognize several limitations of our work. First, our computational model is based on data both from the literature and an *in vitro* coculture system. Thus, it is likely that some aspects of macrophage behavior during the infection, such as response to extracellular signals relevant to iron regulation, are not captured *in vitro*. This is partially addressed by supplementing the experimental data with extensive data from the literature. Second, the present work only pertains to the behavior of monocyte-derived macrophages recruited to the site of infection and does not capture the behavior of other cells (e.g., alveolar macrophages and epithelial cells) relevant to iron handling during aspergillosis. Third, the current model pertains only to the interaction of macrophages with resting and swollen conidia in the first hours of the infection, and not subsequent interactions with fungal hyphae. Finally, Aspergillus itself has a sophisticated system of iron acquisition from the environment, even under iron-starvation conditions. As such, the present work characterizes only part of the complex landscape of iron regulation during aspergillosis.

The current work has several implications for future studies, including suggesting several hypotheses for further exploration: First, as noted above, the mechanisms by which TfR1 is induced in macrophages independent of inflammatory cytokines, and the relevance of this induction to antifungal host defenses, are of interest. Second, the model simulation of BDH2 predicted an increased expression at the steady state due to its regulation by IRP, whereas experimental data showed BDH2 to be downregulated during infection (Fig. 6C and D), suggesting that the known biology of regulation of BDH2 (and, by extrapolation, the role of the mammalian siderophore 2,5-DHBA) during fungal infection is incomplete. Third, the model that we have generated may be useful for predicting outcomes of iron-centered therapeutics, such as pharmacologic iron chelation (15, 105), by providing a better understanding of tissue macrophage iron handling during invasive aspergillosis. Finally, the current model can be incorporated into a multiscale computational model that incorporates the responses of other cell types with the fungus, synthesizing the available data in a systematic way and serving as an *in silico* laboratory.

## MATERIALS AND METHODS

### Ethics statement.

This study was conducted in accordance with the Declaration of Helsinki under a protocol approved by University of Florida Institutional Review Board.

### Fungal culture and harvest.

Aspergillus fumigatus strain 13073 (American Type Culture Collection, Manassas, Virginia) was cultured on Sabouraud’s dextrose agar plates at 37°C for 14 days. Conidia were collected in phosphate-buffered saline (PBS) containing 0.1% Tween 80, filtered through sterile gauze, centrifuged at 700 g, and resuspended in PBS; concentration determined under a hemacytometer.

### Monocyte isolation, culture, and RNA extraction.

Buffy coats from healthy volunteers (ages 21–78, 3 male and 3 female) were purchased (LifeSouth Community Blood Center, Gainesville, FL). Mononuclear cells were isolated using Ficoll gradients and stored at −80°C degrees. CD14^+^ CD16^–^ monocytes were isolated using magnetic negative selection (EasySep Human Monocyte isolation kit, StemCell Technologies, Cambridge, MA) and differentiated into macrophages by culture in RPMI 1640 (Lonza, Morristown, NJ) supplemented with 2 mM l-glutamine, 1 mM sodium pyruvate solution, 0.1 mM nonessential amino-acids, 1% penicillin-streptomycin, 10% fetal bovine serum (HyClone, Logan, UT), and 10 ng/mL recombinant human macrophage colony-stimulating factor (Peprotech, East Windsor NJ) for 7 d. Flow cytometry was used to assess the purity of macrophages after 7 d of culture (Fig. S1), according to a previously published protocol (106), using antibodies against CD68-BV711 (clone Y1/82A) and CD163-FITC (clone GHI/61), purchased from BD Biosciences (San Jose, CA). Macrophages were cocultured with Aspergillus conidia at a 1:1 ratio. At the beginning of the coculture (time zero) and after 2, 4, 6, and 8 h, cells were lysed (RLT buffer, Qiagen, Valencia, CA) and RNA was extracted (RNeasy Plus minikit, Qiagen) following the manufacturer’s instructions.

10.1128/msphere.00074-22.2 FIG S1 Representative flow cytometry plots showing purity of macrophages after culture for 7 days. Download FIG S1, TIF file, 1.2 MB. Copyright © 2022 Adhikari et al. 2022 Adhikari et al. https://creativecommons.org/licenses/by/4.0/ This content is distributed under the terms of the Creative Commons Attribution 4.0 International license.

### RNA-seq library preparation, sequencing, and analysis.

RNA-seq libraries were prepared and sequenced at the Jackson Laboratory for Genomic Medicine. Libraries were generated with the KAPA-Stranded mRNA-seq kit (Roche Sequencing, Wilmington, MA) according to manufacturer’s instructions. Briefly, poly-A RNA was isolated from 300 ng total RNA using oligo-dT magnetic beads. Purified RNA was then fragmented at 85° C for 6 min, targeting fragments ranging 250–300 bp. Fragmented RNA was reverse transcribed with an incubation of 25° C for 10 min, 42° C for 15 min and an inactivation step at 70° C for 15 min, followed by second strand synthesis at 16° C for 60 min. Double stranded cDNA fragments were purified using Ampure XP beads (Beckman Coulter Life Sciences, Indianapolis, IN). The dscDNA were then A-tailed, and ligated with Illumina unique adaptors (Illumina, San Diego, CA). Adaptor-ligated DNA was purified using Ampure XP beads, 10 cycles of PCR amplification, and impurities were eliminated (Ampure XP beads, Beckman Coulter). RNA sequencing was performed on a HiSeq 4000 instrument (Illumina).

The sequenced raw RNA-seq reads were processed to generate read counts for alignment. In brief, the reads were checked for quality control using FASTQC v0.11.8 (www.bioinformatics.babraham.ac.uk/projects/fastqc/), and trimmed using Trimmomatic v0.39 using LEADING:3 TRAILING:3 SLIDINGWINDOW:4:15 MINLEN:36 and a predefined adapter list to be clipped from reads (107). FastQC and Trimmomatic were repeated until desired reads were obtained. MultiQC v1.7 was used to combine individual FastQC results for visualization (108). The trimmed reads were then aligned to the Ensembl GRCh38v96 reference genome using STAR v2.7.2b, and Qualimap v2.2.1 was used to check the quality of alignment (109, 110). Read-count matrix was created by using the column with reads for reverse strandedness (i.e., column 4) from readspergene.tab STAR output files. Samples from one donor were excluded from further analysis based on principal-component analysis (PCA) performed on all samples, showing that this donor’s cells whether incubated alone or with conidia, formed a separate cluster from all other samples, with high biological variation (PC1 38%) relative to other donors and little response (PC2 20%) to the fungus (Fig. S2). PCA was performed on the top 500 variable genes in the data sets using the “prcomp” function in R. Only genes expressed at 10 read counts or higher in at least 5 samples were processed further for differential expression analysis. DESeq 2 v1.24.0 was used to compute differentially expressed genes. The design matrix was created with 5 donors, 1 baseline, and 4 time points for control and coculture groups each. Pairwise comparisons with Wald test (alpha = 0.05) were performed for differential expression analysis. For computational validation of the model using RNA-seq data, RNA-seq read-counts were normalized by the library size and a value of 0.5 was added to the normalized counts to generate pseudo counts, which were then transformed with a Log_2_ scale. Log scaled reads are plotted against time and actual raw read counts, and the line was fitted with loess regression.

10.1128/msphere.00074-22.3 FIG S2 Principal component analysis plot of read counts after variance stabilizing transformation. Conditions are denoted by symbol color and donors by symbol type. Download FIG S2, TIF file, 2.5 MB. Copyright © 2022 Adhikari et al. 2022 Adhikari et al. https://creativecommons.org/licenses/by/4.0/ This content is distributed under the terms of the Creative Commons Attribution 4.0 International license.

Gene Ontology analysis (111, 112) and Reactome (113) enrichment analysis was performed using the clusterProfiler (114, 115) package in R. In these exploratory analyses, we used a threshold of |Log_2_ fold change| ≥ 0.5 (with an adjusted *P* value <0.001) against Homo sapiens background at each time point to identify the top differentially expressed genes and pathways, reasoning that activation of multiple genes in a pathway at this threshold likely represents biological relevance. Gene Ontology terms and Reactome pathways at Benjamini-Hochberg-adjusted *P* values < 0.05 threshold were considered enriched. Ingenuity Pathway Analysis (www.qiagenbio-informatics.com/products/ingenuity-pathway-analysis) was performed to evaluate differentially expressed genes involved in major immune pathways in macrophages with the differentially expressed gene set (4h, 6h, 8h combined). To identify genes involved in iron regulation, we obtained “genes and gene predictions” from AmiGO2 (116) (amigo.geneontology.org/amigo), selecting “iron”.

### Quantitative reverse-transcription PCR and ELISA.

Macrophages were generated and co-incubated with Aspergillus as detailed above. After the incubation period, cells were suspended in RLT buffer (Qiagen, Valencia, CA), homogenized, passed through Qiashredder (Qiagen), and total RNA extracted using the RNAeasy minikit (Qiagen) following the manufacturer’s instructions. Then, 1-0.2 μg of RNA was used to synthesize cDNA using the iScript cDNA synthesis kit (Bio-Rad, Hercules, CA). The cDNA template was mixed with iTAQ SYBR green universal super mix (Bio-Rad), and quantitative PCR was carried out on a CFX Connect system (Bio-Rad). Pre-designed human gene primers were purchased from Bio-Rad (Table S1). Human PPIA was amplified in parallel and used as the reference gene in quantification. Data are expressed as relative gene expression and were calculated using the 2^−ΔΔCT^ method. ELISA were performed on culture supernatants per manufacturer’s instructions (R&D Systems, Minneapolis, MN).

10.1128/msphere.00074-22.1 TABLE S1 PCR primer sequences used in the study. See https://bioradtech.com/data/lookup.php. Download Table S1, DOCX file, 0.01 MB. Copyright © 2022 Adhikari et al. 2022 Adhikari et al. https://creativecommons.org/licenses/by/4.0/ This content is distributed under the terms of the Creative Commons Attribution 4.0 International license.

### Mathematical model formulation and simulation.

From the AmiGO2 database, known iron-related genes were extracted. For the mathematical model, we used a more stringent threshold of |Log_2_ fold change| ≥ 1 (with adjusted *P* value <0.001) to identify the top differentially expressed iron genes from our transcriptional analysis for further consideration. A select few molecules that were not detected as differentially regulated in our transcriptional analysis but are reported as important in macrophage iron regulation in the literature were also considered. We reviewed literature on these molecules and built a static network depicting the relationship (regulatory edges of the network) between the molecules (nodes of the network) in Fig. 4B The static network is the basis for a time- and state-discrete dynamic model, with each node taking on three possible states: 0 (low), 1 (medium), 2 (high). We constructed transition functions encoding regulation of nodes and their evolution in discrete time steps (Table 2). A possible model artifact is a variable change of more than one level per time step, e.g., from low to high without passing through medium. To avoid this, we applied a standard correction that forces this “continuity” property (117), which is known to not affect model features relevant to this study.

### Statistical analyses.

Statistical analyses of RNA-seq data are described above. Other data were analyzed using the Prism software package (version 9.2.0, GraphPad Software, San Diego, California). The area-proportional Euler diagram was generated with EulerAPE (version 3.0.0; open source, http://www.eulerdiagrams.org/eulerAPE/). Comparisons of two groups over time or range of inocula was achieved using two-way ANOVA with Sidak multiple comparison test, and comparison of samples from the same donor under different conditions were performed using Wilcoxon test for paired samples. A *P* value of <0.05 was considered statistically significant. In multiple comparison tests, multiplicity adjusted *P* values are reported.

### Data and code availability.

The data sets and scripts used in this study are made publicly available. The simulation code, in Python 3, and the RNA-seq data in the form of a read-count matrix are available at: https://github.com/NutritionalLungImmunity/NLI_macrophage_iron_regulation. RNA-seq data is deposited to NCBI’s Gene Expression Omnibus and is accessible through GEO Series accession number GSE202286.
