# Supplementary figures and images for: Computational Modeling of Macrophage Iron Sequestration during Host Defense against Aspergillus
Source: mSphere. 2022 Jul 12;7(4):e00074-22. doi: 10.1128/msphere.00074-22 (PMC9429928; doi:10.1128/msphere.00074-22)

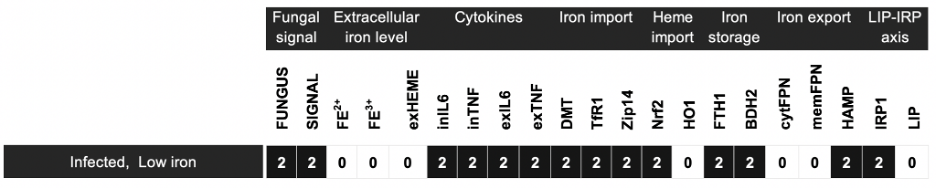

Supplement: FIG S3 [file msphere.00074-22-s0003.tif]

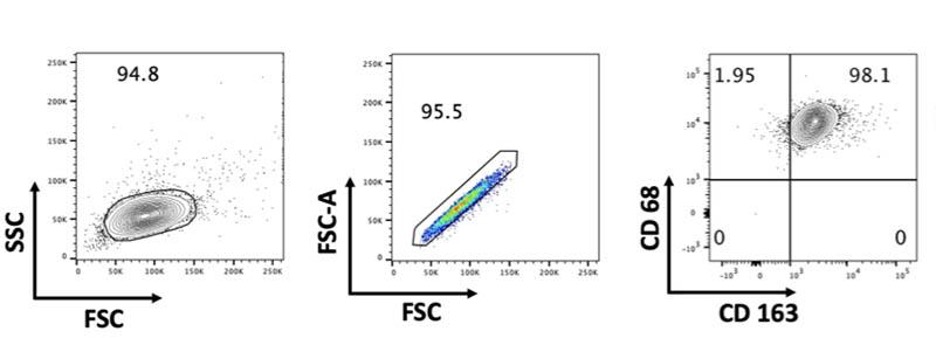

Supplement: FIG S1 [file msphere.00074-22-s0001.tif]

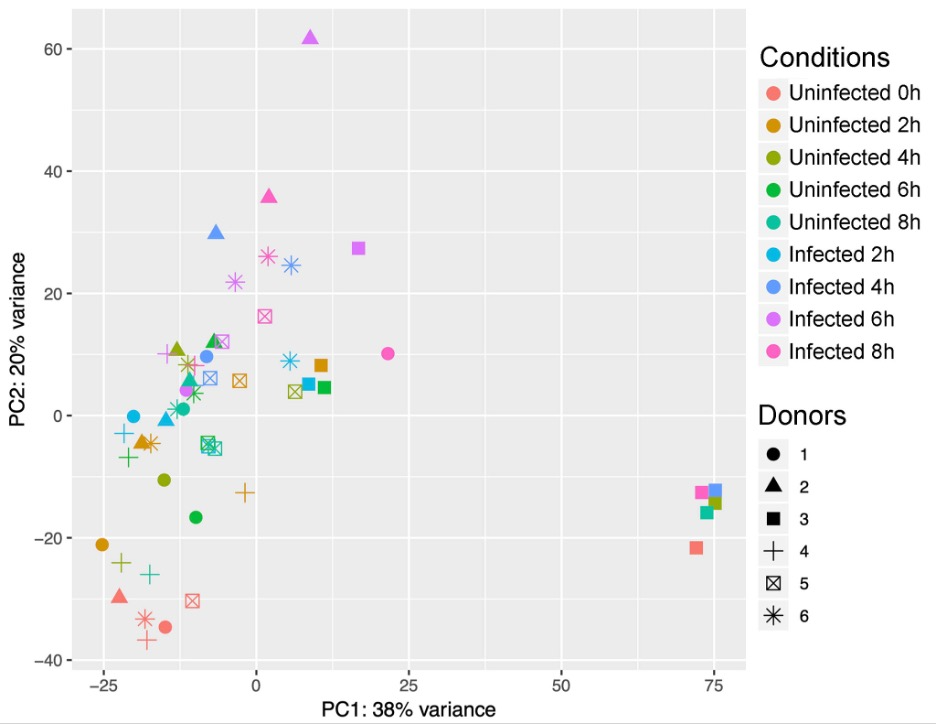

Supplement: FIG S2 [file msphere.00074-22-s0002.tif]
