# Supplementary material for: Computational Modeling of Macrophage Iron Sequestration during Host Defense against Aspergillus
Source: mSphere. 2022 Jul 12;7(4):e00074-22. doi: 10.1128/msphere.00074-22 (PMC9429928; doi:10.1128/msphere.00074-22)
Supplement: TABLE S1 [file msphere.00074-22-s0004.docx]

| **Gene** | **Unique Assay ID (BioRad Prime PCR)** |
| --- | --- |
| BDH2 | qHSACED0046162 |
| FtH1 | qHsaCED0038139 |
| FTL | qHsaCED0057482 |
| IL-6 | qHsaCED0044677 |
| PPIA | qHsaCED0038620 |
| Slc11a2 | qHsaCID0012864 |
| Slc39a8 | qHsaCID0008870 |
| Slc39a14 | qHsaCED0002314 |
| Slc40a1 | qHsaCED0005662 |
| Tfr1 | qHsaCID0022106 |
| TNF | qHsaCED0037461 |
